# Perioperative Outcomes of Neoadjuvant Immunochemotherapy for Locally Resectable Oesophageal Squamous Cell Carcinoma in Geriatric Patients Aged 70 Years or Older

**DOI:** 10.3390/cancers18081192

**Published:** 2026-04-08

**Authors:** Qi Li, Song Lu, Yi Wang, Guangyuan Liu, Zhenjun Liu

**Affiliations:** 1 Department of Critical Care, Sichuan Clinical Research Center for Cancer, Sichuan Cancer Hospital & Institute, Sichuan Cancer Center, University of Electronic Science and Technology of China, Chengdu 610041, China; liqi@scszlyy.org.cn (Q.L.); lusong@scszlyy.org.cn (S.L.); 2 Radiation Oncology Key Laboratory of Sichuan Province, Department of Radiation Oncology, Sichuan Clinical Research Center for Cancer, Sichuan Cancer Hospital & Institute, Sichuan Cancer Center, University of Electronic Science and Technology of China, Chengdu 610041, China; wangyi@scszlyy.org.cn; 3 Department of Thoracic Surgery, Sichuan Clinical Research Center for Cancer, Sichuan Cancer Hospital & Institute, Sichuan Cancer Center, University of Electronic Science and Technology of China, Chengdu 610041, China

**Keywords:** oesophageal squamous cell carcinoma, geriatric patients, neoadjuvant immunochemotherapy, neoadjuvant chemoradiotherapy, postoperative outcomes

## Abstract

**Background:** Neoadjuvant chemoradiotherapy (nCRT) followed by surgery has become the standard treatment for oesophageal cancer. However, data on the outcomes of neoadjuvant immunochemotherapy (nICT) in geriatric patients (≥70 years) who face higher perioperative risks are limited. **Objective:** This study aimed to compare the perioperative outcomes of nICT versus nCRT in elderly patients with locally advanced oesophageal squamous cell carcinoma (ESCC). **Method:** This retrospective cohort study included 132 geriatric patients (median age: 72 years) treated with nICT (n = 51) or nCRT (n = 81) followed by esophagectomy at Sichuan Cancer Hospital (2021–2024). Intraoperative outcomes, postoperative pathologic stages, and complications, including pneumonia and anastomotic leakage, were assessed. Propensity score matching (PSM), overlap weighting (OW), and inverse probability of treatment weighting (IPTW) were used to adjust for baseline covariate imbalances in the sensitivity analysis. **Results:** Pathologic ypT0 stage tended to be higher in the nCRT group (*p* = 0.014), whereas ypN0 was higher in the nICT group (*p* = 0.035). No significant differences in intraoperative or postoperative outcomes between the two groups, except for pulmonary complications (*p* > 0.05). Compared with nCRT patients, nICT patients had significantly lower pulmonary complication rates (13.7% vs. 32.1%, *p* = 0.030), and multivariable analysis confirmed these findings (adjusted OR = 0.26; 95% CI: 0.08–0.85; *p* = 0.026). Sensitivity analyses showed consistent results. **Conclusions:** The safety of nICT is comparable to that of nCRT in geriatric ESCC patients, with significantly fewer pulmonary complications. These findings support nICT as a valuable alternative for elderly populations.

## Simple Summary

Oesophageal cancer is highly prevalent in elderly patients, yet data on neoadjuvant immunochemotherapy for patients aged 70 and older with resectable oesophageal squamous cell carcinoma (ESCC) are limited. In this study, the perioperative outcomes of 132 geriatric patients who received neoadjuvant immunochemotherapy (nICT) and chemoradiotherapy (nCRT) were compared, and the results revealed that the two treatments had comparable intraoperative outcomes and most postoperative outcomes. Notably, nICT reduced the risk of pulmonary complications and increased the rate of pathological response to lymph nodes, whereas the primary tumour response to chemoradiotherapy was greater. These results support nICT as a safe, valuable treatment alternative for elderly ESCC patients, guiding clinical treatment decisions for this vulnerable population.

## 1. Introduction

Oesophageal cancer ranks as the fifth most common cause of cancer-related mortality globally, imposing a significant public health burden [1]. In China, it constitutes the fourth most common cause of cancer-related death [2], with oesophageal squamous cell carcinoma (ESCC) accounting for more than 90% of cases [3]. Neoadjuvant chemoradiotherapy (nCRT) followed by radical resection has become the standard of care for patients with locally advanced ESCC [4]. However, evidence indicates that the recurrence rates among ESCC patients treated with this regimen exceed 30% within 3–5 years [4,5], underscoring the need for improved therapeutic strategies.

Recently, neoadjuvant immunochemotherapy (nICT) followed by surgery has emerged as a promising approach for reducing recurrence and enhancing long-term survival for patients with ESCC. Several studies have demonstrated that, compared with nCRT, nICT yields comparable or even superior perioperative outcomes in this patient population [6,7].

With the ageing of the population in China and the West, along with increasing life expectancy, the number of older patients diagnosed with oesophageal cancer has increased significantly. Geriatric patients often present with an increased risk of multiple comorbidities and frailty, which are associated with adverse postoperative outcomes after esophagectomy [8,9]. However, previous investigations have largely overlooked this subgroup, with limited inclusion of patients aged 70 years or older. Multiple studies have reported that elderly patients (≥70 years) face an increased risk of post-esophagectomy mortality [10,11,12]. Consequently, the applicability of existing findings on nICT and nCRT to this vulnerable population remains unclear.

This study aims to address this knowledge gap by comparing the perioperative outcomes of elderly patients with locally advanced ESCC who received nICT versus those who received nCRT followed by esophagectomy. By examining this specific cohort, our study seeks to inform treatment decisions and optimize outcomes for geriatric patients with ESCC.

## 2. Methods

### 2.1. Study Design and Participants

**Figure 1:**
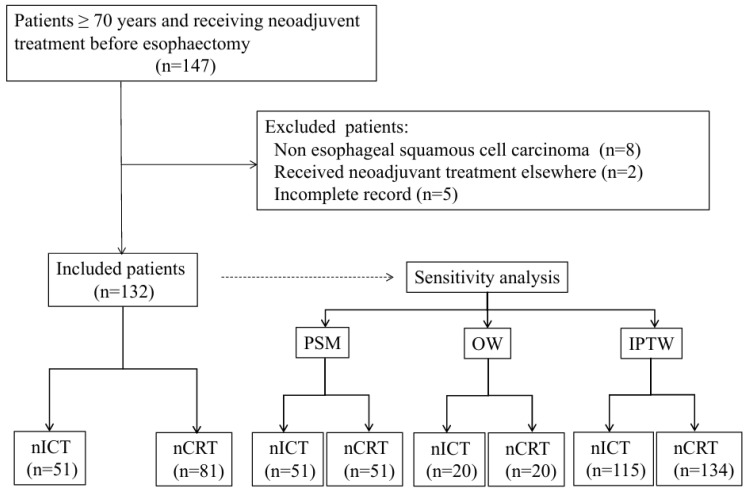
Flowchart of patient enrolment. nICT, neoadjuvant immunochemotherapy; nCRT, neoadjuvant chemoradiotherapy; PSM, propensity score matching; OW, overlap weighting; IPTW, inverse probability of treatment weighting.

In accordance with the ethical review board of the hospital (SCCHEC-02-2023-029), this retrospective cohort study included patients aged ≥70 years with ESCC who underwent nICT or nCRT followed by radical esophagectomy at Sichuan Cancer Hospital between January 2021 and October 2024. Informed consent was waived due to the retrospective nature of the study. The exclusion criteria included non-ESCC histology, incomplete medical records, and the use of neoadjuvant treatment at external institutions. The screening flowchart is shown in Figure 1.

Patient demographics, preoperative characteristics, intraoperative data, and postoperative outcomes were extracted from electronic medical records. The age-adjusted Charlson Comorbidity Index (CCI) was used to assess the severity of comorbid disease [13]. Clinical and postoperative pathologic tumour stage (ypTNM) was classified according to the eighth edition of the AJCC/UICC criteria [14].

### 2.2. Neoadjuvant Treatment and Esophagectomy

All participants received 2–4 cycles of neoadjuvant treatment. For the nICT group, immunotherapy was administered as a PD-1 inhibitor at the following standard doses: 240 mg of toripalimab, 200 mg of camrelizumab, 200 mg of sintilimab, 100–200 mg of pembrolizumab, or 360 mg of nivolumab combined with chemotherapy every 21 days. The predominant chemotherapy regimen was platinum-taxane (paclitaxel 175 mg/m^2^ plus cisplatin/nedaplatin 80 mg/m^2^ or carboplatin AUC = 5), with a platinum–fluorouracil regimen (platinum agents 75–80 mg/m^2^ plus fluorouracil 800 mg/m^2^) as an alternative. For the nCRT group, chemotherapy was delivered weekly with concurrent radiotherapy: the main regimen was 25 mg/m^2^ cisplatin or a carboplatin AUC = 2 plus 50 mg/m^2^ paclitaxel (or the alternative platinum–fluorouracil regimen), and radiotherapy was performed at a total dosage ranging from 37.6 to 50.2 Gy in 1.8 to 2.0 Gy fractions over 10 to 33 sessions (5 fractions/week). To protect normal lung tissue, predefined dose constraints were strictly applied: lung V5 ≤ 60%, V20 ≤ 30%, V30 ≤ 20%, and mean lung dose (MLD) < 18 Gy. The therapeutic regimens were decided by the physicians and patients on the basis of the patient’s choice and physical state.

Surgery was scheduled 4–8 weeks after the final neoadjuvant treatment session in the absence of surgical contraindications. The surgical options were selected on the basis of performance status and tumour location and extent. The surgical approaches included McKeown or Ivor–Lewis esophagectomy via da Vinci robot-assisted esophagectomy, minimally invasive esophagectomy (MIE), transthoracic open esophagectomy, or the conversion method (from video-assisted thoracoscopic with laparoscopic to open operations). Lymphadenectomy was routinely conducted.

### 2.3. Postoperative Complications

Postoperative complications were graded according to the Clavien–Dindo (CD) classification, with grade 3 or higher defined as major complications [15]. Pulmonary complications included pneumonia, pleural effusion, acute respiratory distress syndrome (ARDS), pneumothorax, and empyema. Pneumonia was diagnosed on the basis of clinical and radiographic criteria. Anastomotic complications included anastomotic leakage, conduit necrosis, anastomotic stricture, and anastomotic inflammation. Anastomotic leakage was diagnosed by the presence of contrast extravasation detected via radiographic examination or endoscopy. Other complications included arrhythmias requiring pharmacological treatment or cardioversion, severe diarrhoea, incision infection, and septic shock.

### 2.4. Statistical Analysis

Statistical analyses were performed using R (R Foundation for Statistical Computing Platform, version 4.4.0). Quantitative variables are presented as medians with interquartile ranges (25th quartile–75th quantile) or means ± SDs (standard deviations). Comparisons between two groups were conducted using the Wilcoxon test or Student’s *t* test, as appropriate. The Kolmogorov–Smirnov test was used to test the normal distribution. Categorical variables are expressed as frequencies (percentages) and were compared using Pearson’s chi-square test with Yates’ continuity correction or Fisher’s exact test.

Univariate and multivariate logistic regression analyses were employed to assess the impact of neoadjuvant treatment on postoperative complications, with all preoperative (baseline) variables incorporated into the multivariate analysis via backward stepwise selection, removing variables with *p* ≥ 0.05. Due to the unbalanced sample size and baseline covariates between the two groups, propensity score matching (PSM, 1:1 k-nearest neighbour matching without preset callipers), propensity score-based overlap weighting (OW), and inverse probability of treatment weighting (IPTW) were utilized for sensitivity analysis. The propensity score was estimated using a logistic regression model including all baseline covariates. A standardized mean difference (SMD) ≤ 0.1 was considered indicative of covariate balance. All *p* values were two-sided, and *p* < 0.05 was considered statistically significant.

## 3. Results

### 3.1. Baseline Characteristics

A total of 132 patients were included, with a median age of 72 years (83.3% male). The demographic, CCI, preoperative neutrophil, lymphocyte, haemoglobin, and albumin levels, as well as cardiac function (ejection fraction), pulmonary function, American Society of Anaesthesiologists (ASA) Physical Status Classification, clinical tumour stage, and tumour location, are shown in Table 1. Group comparisons revealed significant differences in smoking history, CCI score, lymphocytes, FVC% (forced vital capacity percent of the predicted), FEV1% (forced expiratory volume in 1 s percent of the predicted), and clinical N stage (*p* < 0.05).

### 3.2. Intraoperative and Postoperative Outcomes

The majority of patients received MIE (81.8%) and the McKeown procedure (92.4%). Significant differences in ypT (*p* = 0.014) and ypN stages (*p* = 0.035) were observed between the nICT and nCRT groups (Table 2). No between-group differences were observed in terms of surgical approach, procedure type, surgery time, estimated blood loss, blood transfusion, postoperative haemoglobin and albumin, pCR, ypTNM, continuous renal replacement therapy (CRRT), ventilator use in the ICU, ICU stay, hospital length of stay (LOS), 24 h reoperation, 24 h ICU readmission, 30-day ICU readmission, 30-day rehospitalization, or 30-day mortality (all *p* > 0.05).

### 3.3. Postoperative Complications

Forty-five patients (34.1%) developed postoperative complications, and pulmonary complications accounted for the majority (25.0%) of the complications. The nICT group had a significantly lower rate of pulmonary complications than the nCRT group (13.7% vs. 32.1%, *p* = 0.030), as shown in Table 3. The specific diagnoses of pulmonary complications are illustrated in Figure S1. Univariable and multivariable logistic analyses confirmed this association (crude OR = 0.34, 95% CI = 0.13–0.85; *p* = 0.021; adjusted OR = 0.26, 95% CI = 0.08–0.85; *p* = 0.026; Table 4).

Furthermore, we observed a reduced occurrence of pneumonia (3.9% vs. 18.5%, *p* = 0.030) and a crude OR = 0.18 (95% CI, 0.04–0.82; *p* = 0.027), although this difference was not significant in the multivariable analysis (adjusted OR, 95% CI, 0.21, 0.03–1.52; *p* = 0.123). Anastomotic leakage rates did not differ between the two groups (3.9% vs. 9.9%, *p* = 0.315).

All complications, major all complications, major pulmonary complications, anastomotic complications, major anastomotic complications, other complications, and major other complications were comparable (all *p* > 0.05; Table 3 and Table 4).

### 3.4. Sensitivity Analysis

**Figure 2:**
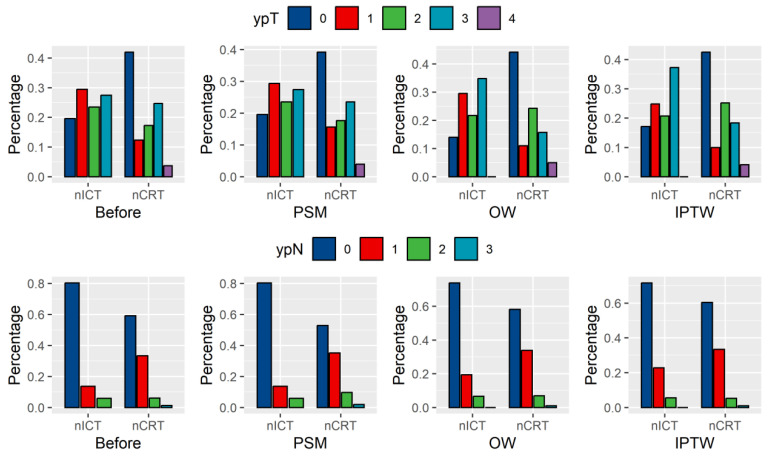
Proportion of ypT0 and ypN0 in the nICT and nCRT. nICT, neoadjuvant immunochemotherapy; nCRT, neoadjuvant chemoradiotherapy.

PSM, OW, and IPTW achieved covariate balance, and the distributions of baseline covariates after matching or weighing are shown in Tables S1–S3. A trend towards higher ypT0 proportions in patients receiving nICT was observed (Figure 2), although significance was not always achieved (*p* = 0.089 after PSM, *p* = 0.088 after OW, *p* < 0.001 after IPTW). However, the proportion of patients who received nICT was greater for ypN0 (*p* = 0.015 after PSM, *p* = 0.731 after OW, *p* = 0.164 after IPTW).

Consistent with these findings, significantly fewer postoperative pulmonary complications were observed in patients who underwent nICT after PSM (adjusted OR, 95% CI: 0.17, 0.03–0.82; *p* = 0.027), after OW (adjusted OR, 95% CI: 0.26, 0.07–0.88; *p* = 0.031), and after IPTW (adjusted OR, 95% CI: 0.30, 0.11–0.79, *p* = 0.015). The results are shown in Table S4.

nICT did not lead to an increased risk of postoperative complications, including pneumonia and anastomotic leakage, as shown after PSM, OW, and IPTW (*p* > 0.05 for all; Table S4).

## 4. Discussion

Accumulating evidence reveals that elderly patients with locally advanced oesophageal cancer can benefit from multimodal therapy, including esophagectomy, chemotherapy, and radiotherapy [16,17,18]. Esophagectomy remains the mainstay of treatment for localized oesophageal cancer, and immunotherapy has emerged as a promising treatment because of its favourable efficacy and safety profile [19]. To date, research investigating the perioperative outcomes of nICT followed by esophagectomy in elderly patients (≥70 years) remains limited.

In the nCRT group, a greater proportion of patients had ypT0 disease, which is consistent with the findings of prior studies [7,20,21]; this finding likely reflects the potent local control of primary lesions by radiotherapy. Conversely, the nICT group had a greater proportion of ypN0 patients, which is consistent with the systemic anti-tumour mechanism of immunotherapy and may confer advantages in controlling metastatic lymph node disease. However, the literature on the impact of nICT on ypN0 is inconsistent, with some studies reporting increased [21] or decreased ypN0 rates [20,22] compared with those of nCRT. Moreover, significance was not consistently maintained after the propensity score-based adjustments were made, suggesting that unmeasured covariates may influence postoperative pathological staging. Ultimately, differences in ypN0 and ypT0 stages were detected between nICT and nCRT, whereas no significant differences were detected in ypTNM stage or pCR. The potential differences in the long-term prognosis between nICT and nCRT in geriatric ESCC patients require clarification through rigorously designed larger prospective trials.

Postoperative pulmonary complications are the most common morbidities after oesophagectomy and are associated with increased mortality and decreased health-related quality of life [23]. Advanced age is an independent risk factor for such complications after esophagectomy [24], likely due to age-related physiological declines, including postoperative diaphragmatic dysfunction [25], sarcopenia [26], and impaired pulmonary function [27]. Our study revealed that compared with nCRT, nICT was associated with fewer pulmonary complications in older patients, a finding that was sustained after multivariable adjustment and sensitivity analyses.

Prior studies in mixed-age ESCC cohorts reported comparable pulmonary complication rates between nICT and nCRT [7,21,22]. Theoretically, adding radiotherapy to chemotherapy may increase complication risks due to radiation-induced tissue injury near the oesophagus. However, clinical research investigating the impact of nCRT on pulmonary complications is debated: one meta-analysis linked nCRT to increased pulmonary complications compared with neoadjuvant chemotherapy [28], whereas another found no difference [29]. This heterogeneity may stem from variations in radiotherapy techniques, surgical approaches, chemotherapy regimens, or patient populations. Two recent insightful studies highlight that patients with reduced lung volumes or preexisting pulmonary disease—more prevalent in older adults—are at higher risk of pulmonary complications [30,31]. nICT does not cause radiation-induced lung injury, and these findings underscore the clinical relevance of our results.

Pneumonia and anastomotic leakage are among the most critical postesophagectomy complications [32]. Our data revealed that compared with nCRT, nICT did not increase the risk of these events in elderly ESCC patients, which is consistent with the findings of prior studies [21] and a meta-analysis [33] in mixed-age patients. Notably, univariable analysis revealed a significantly lower pneumonia rate in the nICT group, although this difference was attenuated in multivariate analysis. Yang GZ et al. [34] reported a similar trend in younger patients (≤60 years, 50.5%), where compared with nCRT, nICT was associated with a lower pneumonia incidence (5.70% vs. 14.58%, *p* = 0.062), suggesting potential benefits of nICT.

To date, several novel neoadjuvant immunotherapy regimens have been investigated in trials for patients with resectable locally advanced ESCC. A prospective randomized phase III clinical trial enrolling 252 patients revealed that compared with neoadjuvant chemotherapy, nICT combined with postoperative immunotherapy (toripalimab) resulted in a higher pCR rate (18.6% vs. 4.6%) and improved the 1-year event-free survival (EFS) rate (77.9% vs. 64.3%) and 1-year overall survival (OS) rate (94.1% vs. 83.0%) [35]. The rates of postoperative Clavien–Dindo grade and morbidity did not differ between the two groups. An open-label, single-arm, single-institution phase II trial including 21 patients receiving neoadjuvant immunotherapy (tislelizumab) with chemoradiotherapy showed promising efficacy (pCR rate 52.6%, 3-year DFS 65.2%, and OS 95.2%) and a manageable safety profile [36]. A single-arm, phase 1b trial of neoadjuvant PD-L1 blockade with adebrelimab provided a potential rationale for neoadjuvant anti-PD-L1 monotherapy with a pCR rate of 8%, a 2-year OS of 92%, and acceptable adverse events [37]. These studies were not designed for elderly patients, especially those aged 70 years or older. Although perioperative immunotherapy, neoadjuvant chemoradiotherapy combined with immunotherapy, and neoadjuvant PD-L1 blockade may improve treatment efficacy, they might increase perioperative risks in this population. Our study demonstrates the perioperative safety of neoadjuvant immunochemotherapy in elderly patients aged 70 years or older, highlighting the need for more research on the perioperative risk assessment of these novel immunotherapeutic regimens.

Our research has several limitations. First, this was a retrospective study with a modest sample size, and the treatment allocation was not randomized; although multiple covariate balance methods were applied, potential biases such as selection bias could not be eliminated. Therefore, our findings should be interpreted with caution. A prospective randomized trial is needed to validate these findings. Second, we focused solely on short-term postoperative outcomes; the differences in survival between the mid-term and long-term outcomes need further investigation. Third, the immunotherapy, radiotherapy, and chemotherapy administered varied across patients, which mirrored real-world practice but limited the ability to derive definitive conclusions. Fourth, this study did not include comprehensive geriatric assessment tools or performance status scales, which may limit the generalizability of our findings. Finally, caution is advised when these results are generalized to nonsquamous histological subtypes, as the cohort included only ESCC patients.

## 5. Conclusions

Compared with nCRT, nICT was associated with a higher proportion of ypN0 and a lower proportion of ypT0. The two groups did not significantly differ in terms of the majority of postoperative complications. Notably, nICT was linked to a reduced incidence of pulmonary complications. Importantly, nICT did not increase the risk of anastomotic leakage and tended to decrease pneumonia. These findings support the safety profile of nICT in elderly populations.

## Figures and Tables

**Table 1:** Baseline characteristics of the patients.

	Overall	nICT	nCRT	*p* Value	SMD
Variables	(n = 132)	(n = 51)	(n = 81)		
Age (years)	72 (71–74)	73 (71–74)	72 (70–74)	0.244	−0.148
Male gender	110 (83.3%)	39 (76.5%)	71 (87.7%)	0.150	0.295
BMI (kg/m^2^)	23.23 ± 3.06	22.86 ± 2.49	23.47 ± 3.37	0.266	−0.206
Smoking history	68 (51.5%)	19 (37.3%)	49 (60.5%)	0.015	0.478
Drinking history	58 (43.9%)	17 (33.3%)	41 (50.6%)	0.077	0.356
Family history of oesophageal cancer	17 (12.9%)	5 (9.8%)	12 (14.8%)	0.569	0.153
CCI score	6.0 (6.0–7.0)	6.0 (6.0–6.5)	6.0 (6.0–7.0)	0.004	−0.551
Neutrophil (×109/L)	3.01 (2.41–3.84)	3.10 (2.61–4.03)	2.92 (2.25–3.78)	0.226	0.086
Lymphocyte (×109/L)	0.95 (0.69–1.23)	1.17 (0.96–1.39)	0.85 (0.63–1.06)	<0.001	0.701
Platelet (×109/L)	158.50 (131.00–196.75)	167.00 (130.50–199.00)	157.00 (132.00–183.00)	0.650	0.061
Haemoglobin (g/L)	116.94 ± 14.37	115.90 ± 14.64	117.59 ± 14.26	0.513	−0.116
Albumin (g/L)	37.31 ± 3.65	37.97 ± 3.86	36.89 ± 3.46	0.097	0.296
FVC (L)	2.81 ± 0.60	2.89 ± 0.65	2.77 ± 0.57	0.256	0.266
FVC(% pred)	90.25 (81.30–104.38)	97.20 (83.95–108.10)	88.50 (81.00–100.00)	0.043	0.317
FEV1 (L)	2.21 ± 0.49	2.28 ± 0.55	2.16 ± 0.44	0.168	0.241
FEV1 (% pred)	93.60 (84.22–107.60)	97.90 (86.35–113.55)	90.90 (84.00–99.60)	0.032	0.391
FEV1/FVC (% pred)	79.19 (73.05–84.60)	78.69 (74.16–83.81)	80.25 (73.00–84.68)	0.483	−0.079
DLCO (L)	5.96 ± 1.54	6.10 ± 1.48	5.87 ± 1.59	0.414	0.101
DLCO (% pred)	84.10 (71.80–94.70)	83.90 (75.05–93.50)	84.50 (68.60–94.70)	0.695	0.066
EF (%)	68.00 (65.00–70.00)	68.00 (65.50–70.00)	68.00 (65.00–70.00)	0.404	0.169
ASA	2.11 ± 0.34	2.06 ± 0.31	2.15 ± 0.36	0.144	−0.267
Clinical T stage				0.681	0.101
2	3 (2.3%)	1 (2.0%)	2 (2.5%)		
3	113 (85.6%)	42 (82.4%)	71 (87.7%)		
4	16 (12.1%)	8 (15.7%)	8 (9.9%)		
Clinical N stage				0.018	−0.520
0	1 (0.8%)	1 (2.0%)	0 (0.0%)		
1	47 (35.6%)	25 (49.0%)	22 (27.2%)		
2	74 (56.1%)	21 (41.2%)	53 (65.4%)		
3	10 (7.6%)	4 (7.8%)	6 (7.4%)		
Clinical TNM				0.125	−0.308
II	2 (1.5%)	2 (3.9%)	0 (0.0%)		
III	106 (80.3%)	38 (74.5%)	68 (84.0%)		
IV	24 (18.2%)	11 (21.6%)	13 (16.0%)		
Tumour location				0.898	0.083
Upper	23 (17.4%)	9 (17.6%)	14 (17.3%)		
Middle	78 (59.1%)	29 (56.9%)	49 (60.5%)		
Lower	31 (23.5%)	13 (25.5%)	18 (22.2%)		

nICT, neoadjuvant immunochemotherapy; nCRT, neoadjuvant chemoradiotherapy; SMD, standardized mean difference; BMI, body mass index; CCI, Charlson Comorbidity Index; FVC, forced vital capacity; % pred, percent of the predicted; FEV1, forced expiratory volume in 1 s; DLCO, diffusing capacity of the lung for carbon monoxide; EF, ejection fraction; ASA, American Society of Anaesthesiologists Physical Status Classification.

**Table 2:** Intraoperative and postoperative outcomes.

	Overall	nICT	nCRT	*p* Value
Variables	(n = 132)	(n = 51)	(n = 81)	
Surgical approach				0.526
Robot-assisted	10 (7.6%)	3 (5.9%)	7 (8.6%)	
MIE	108 (81.8%)	42 (82.4%)	66 (81.5%)	
Open	12 (9.1%)	6 (11.8%)	6 (7.4%)	
Converted to open	2 (1.5%)	0 (0.0%)	2 (2.5%)	
Procedure type				0.538
Ivor–Lewis	8 (6.1%)	4 (7.8%)	4 (4.9%)	
McKeown	122 (92.4%)	47 (92.2%)	75 (92.6%)	
Others	2 (1.5%)	0 (0.0%)	2 (2.5%)	
Surgery time (hours)	4.08 ± 0.94	4.15 ± 1.12	4.03 ± 0.82	0.466
Estimated blood loss (mL)	177.88 ± 108.33	171.76 ± 115.01	181.73 ± 104.46	0.609
Blood transfusion	10 (7.6%)	6 (11.8%)	4 (4.9%)	0.269
Haemoglobin (g/L)	110.33 ± 16.10	109.39 ± 16.60	110.93 ± 15.85	0.596
Albumin (g/L)	30.59 ± 4.22	31.26 ± 4.56	30.16 ± 3.97	0.148
ypT stage				0.014
T0	44 (33.3%)	10 (19.6%)	34 (42.0%)	
T1	25 (18.9%)	15 (29.4%)	10 (12.3%)	
T2	26 (19.7%)	12 (23.5%)	14 (17.3%)	
T3	34 (25.8%)	14 (27.5%)	20 (24.7%)	
T4	3 (2.3%)	0 (0.0%)	3 (3.7%)	
ypN stage				0.035
N0	89 (67.4%)	41 (80.4%)	48 (59.3%)	
N1	34 (25.8%)	7 (13.7%)	27 (33.3%)	
N2	8 (6.1%)	3 (5.9%)	5 (6.2%)	
N3	1 (0.8%)	0 (0.0%)	1 (1.2%)	
ypTNM stage				0.129
I	78 (59.1%)	32 (62.7%)	46 (56.8%)	
II	16 (12.1%)	9 (17.6%)	7 (8.6%)	
III	35 (26.5%)	10 (19.6%)	25 (30.9%)	
IV	3 (2.3%)	0 (0.0%)	3 (3.7%)	
pCR	38 (28.8%)	10 (19.6%)	28 (34.6%)	0.099
ICU CRRT use	1 (0.8%)	0 (0.0%)	1 (1.2%)	>0.999
ICU ventilator use	9 (6.8%)	1 (2.0%)	8 (9.9%)	0.152
ICU stay (days)	1.3 (1.0,1.5)	1.2 (1.1,1.4)	1.2 (1.0–1.5)	0.083
LOS (days)	13.6 (11.3–17.4)	13.5 (11.3–16.8)	13.4 (11.3–18.3)	0.710
24-Hour reoperation	3 (2.3%)	1 (2.0%)	2 (2.5%)	>0.999
24-Hour ICU readmission	5 (3.8%)	1 (2.0%)	4 (4.9%)	0.648
30-Day ICU readmission	6 (4.5%)	1 (2.0%)	5 (6.2%)	0.405
30-Day rehospitalization	1 (0.8%)	1 (2.0%)	0 (0.0%)	0.386
30-Day mortality	1 (0.8%)	0 (0.0%)	1 (1.2%)	>0.999

nICT, neoadjuvant immunochemotherapy; nCRT, neoadjuvant chemoradiotherapy; MIE, minimally invasive oesophagectomy; pCR, pathological complete regression; CRRT, continuous renal replacement therapy; ICU, intensive care unit; LOS, hospital length of stay.

**Table 3:** Postoperative complications.

	Overall	nICT	nCRT	*p* Value
Variables	(n = 132)	(n = 51)	(n = 81)	
All complications	45 (34.1%)	12 (23.5%)	33 (40.7%)	0.065
Major all complications	32 (24.2%)	9 (17.6%)	23 (28.4%)	0.232
Pulmonary complications	33 (25.0%)	7 (13.7%)	26 (32.1%)	0.030
Major pulmonary complications	23 (17.4%)	5 (9.8%)	18 (22.2%)	0.111
Anastomotic complications	13 (9.8%)	3 (5.9%)	10 (12.3%)	0.361
Major anastomotic complications	12 (9.1%)	3 (5.9%)	9 (11.1%)	0.368
Other complications	8 (6.1%)	5 (9.8%)	3 (3.7%)	0.260
Major other complications	6 (4.5%)	3 (5.9%)	3 (3.7%)	0.676
Pneumonia	17 (12.9%)	2 (3.9%)	15 (18.5%)	0.030
Anastomotic leakage	10 (7.6%)	2 (3.9%)	8 (9.9%)	0.315

nICT, neoadjuvant immunochemotherapy; nCRT, neoadjuvant chemoradiotherapy.

**Table 4:** Logistic regression analysis of postoperative complications.

Complications	Treatment	Crude OR (95% CI)	*p* Value	Adjusted OR (95% CI)	*p* Value
All complications					
	nCRT	Reference			
	nICT	0.45 (0.20–0.98)	0.045	0.47 (0.17–1.29)	0.141
Major all complications					
	nCRT	Reference			
	nICT	0.54 (0.23–1.29)	0.164	0.56 (0.19–1.64)	0.291
Pulmonary complications					
	nCRT	Reference			
	nICT	0.34 (0.13–0.85)	0.021	0.26 (0.08–0.85)	0.026
Major pulmonary complications					
	nCRT	Reference			
	nICT	0.38 (0.13–1.10)	0.074	0.34 (0.09–1.34)	0.123
Anastomotic complications					
	nCRT	Reference			
	nICT	0.44 (0.12–1.70)	0.235	0.20 (0.03–1.18)	0.076
Major anastomotic complications					
	nCRT	Reference			
	nICT	0.50 (0.13–1.94)	0.317	0.28 (0.04–1.71)	0.167
Other complications					
	nCRT	Reference			
	nICT	2.83 (0.65–12.38)	0.168	3.26 (0.33–32.21)	0.313
Major other complications					
	nCRT	Reference			
	nICT	1.62 (0.32–8.38)	0.562	1.72 (0.15–19.14)	0.659
Pneumonia					
	nCRT	Reference			
	nICT	0.18 (0.04–0.82)	0.027	0.21 (0.03–1.52)	0.123
Anastomotic leakage					
	nCRT	Reference			
	nICT	0.37 (0.08–1.83)	0.224	0.21 (0.03–1.66)	0.139

nICT, neoadjuvant immunochemotherapy; nCRT, neoadjuvant chemoradiotherapy; OR, odds ratio; CI, confidence interval.

## Data Availability

The data for this study are available upon request from the corresponding author.

## Supplementary Materials

The following supporting information can be downloaded at https://www.mdpi.com/article/10.3390/cancers18081192/s1; Figure S1: Types of pulmonary complications; Table S1: Baseline characteristics of the patients after PSM; Table S2: Baseline characteristics of the patients after OW; Table S3: Baseline characteristics of the patients after IPTW; Table S4: The logistic regression analysis of postoperative complications after matching/weighting.
