# Supplementary material for: Perioperative Outcomes of Neoadjuvant Immunochemotherapy for Locally Resectable Oesophageal Squamous Cell Carcinoma in Geriatric Patients Aged 70 Years or Older
Source: Cancers (Basel). 2026 Apr 8;18(8):1192. doi: 10.3390/cancers18081192 (PMC13115034; doi:10.3390/cancers18081192)
Supplement: Supplementary file 1 [file cancers-18-01192-s001.zip › cancers-4201930-supplementary.pdf]

cancers

MDPI

Supplementary Materials: Perioperative Outcomes of Neoadjuvant Immunochemotherapy for Locally Resectable Esophageal Squamous Cell Carcinoma in Geriatric Patients Aged 70 Years or Older

Qi Li, Song Lu [1], Yi Wang, Guangyuan Liu and Zhenjun Liu

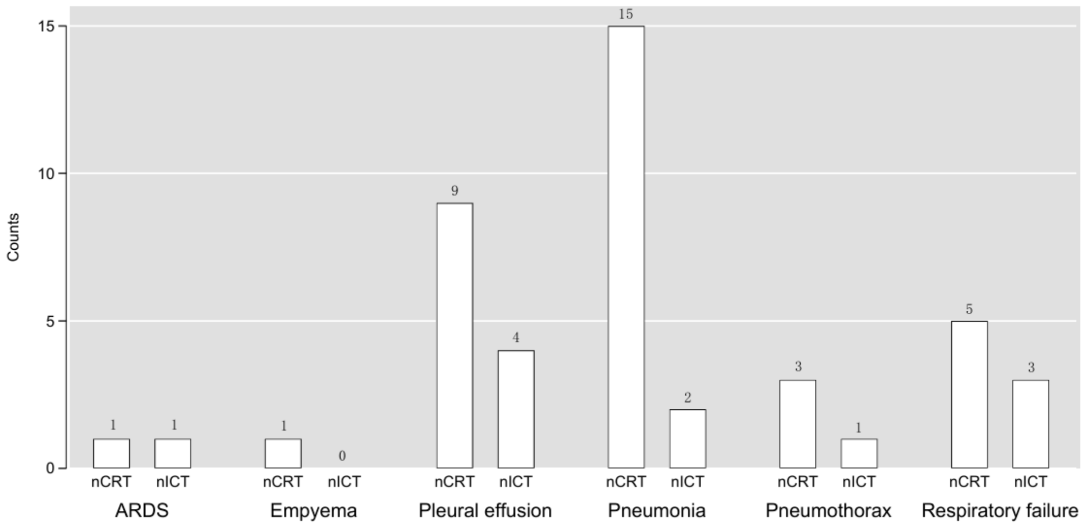

Figure S1. Types of pulmonary complications.

Table S1. Baseline characteristics of the patients after PSM.

	Overall	nICT	nCRT	P value	SMD
Variables	(n=102)	(n=51)	(n=51)		
Age (years)	72.53±2.40	72.45±2.49	72.61±2.32	0.743	-0.063
Male gender	83(81.4%)	39(76.5%)	44(86.3%)	0.309	0.098
BMI (kg/m²)	23.04±2.52	22.86±2.49	23.22±2.57	0.473	-0.145
Smoking history	44(43.1%)	19(37.3%)	25(49.0%)	0.318	0.118
Drinking history	40(39.2%)	17(33.3%)	23(45.1%)	0.311	0.118
Family history of esophageal cancer	12(11.8%)	5(9.8%)	7(13.7%)	0.759	0.039
CCI score	6.00(6.00-7.00)	6.00(6.00-6.50)	6.00(6.00-7.00)	0.225	-0.250
Neutrophil(×10⁹/L)	3.02(2.47-3.85)	3.10(2.61-4.03)	2.92(2.24-3.75)	0.237	0.071
Lymphocyte(×10⁹/L)	1.05(0.80-1.27)	1.17(0.96-1.39)	0.91(0.69-1.08)	0.002	0.477
Platelet(×10⁹/L)	156.50(131.00-198.25)	167.00(130.50-199.00)	149.00(133.00-182.50)	0.705	0.034
Hemoglobin(g/L)	116.18±14.69	115.90±14.64	116.45±14.89	0.851	-0.038
Albumin(g/L)	37.59±3.83	37.97±3.86	37.20±3.80	0.31	0.201
FVC(L)	2.88±0.60	2.92±0.65	2.83±0.55	0.457	0.137
FVC(% pred)	92.20(82.43-106.25)	97.20(83.95-108.10)	89.10(82.20-101.50)	0.203	0.177
FEV1(L)	2.26±0.48	2.28±0.55	2.24±0.40	0.631	0.084
FEV1(% pred)	96.90(85.90-110.30)	97.90(86.35-113.55)	92.80(86.00-107.15)	0.235	0.216
FEV1/FVC(% pred)	79.08(73.26-84.60)	78.69(74.16-83.81)	80.85(72.89-84.80)	0.445	-0.145
DLCO(L)	6.15±1.56	6.12±1.50	6.18±1.62	0.846	-0.040
DLCO(% pred)	84.10(73.12-94.92)	83.90(75.05-93.50)	84.50(68.70-97.35)	0.965	-0.019
EF(%)	68.00(66.00-70.00)	68.00(65.50-70.00)	68.00(66.00-70.00)	0.652	0.082

 https://doi.org/10.3390/cancers18081192

ASA	2.08±0.30	2.06±0.31	2.10±0.30	0.518	-0.126
Clinical T stage				0.886	0.098
2	2(2.0%)	1(2.0%)	1(2.0%)		
3	86(84.3%)	42(82.4%)	44(86.3%)		
4	14(13.7%)	8(15.7%)	6(11.8%)		
Clinical N stage				0.512	-0.275
0	1(1.0%)	1(2.0%)	0(0.0%)		
1	43(42.2%)	24(47.1%)	19(37.3%)		
2	50(49.0%)	23(45.1%)	27(52.9%)		
3	8(7.8%)	3(5.9%)	5(9.8%)		
Clinical TNM stage				0.398	0.000
Ⅱ	2(2.0%)	2(3.9%)	0(0.0%)		
Ⅲ	82(80.4%)	39(76.5%)	43(84.3%)		
Ⅳ	18(17.6%)	10(19.6%)	8(15.7%)		
Tumor location				0.83	0.030
Upper	17(16.7%)	9(17.6%)	8(15.7%)		
Middle	61(59.8%)	29(56.9%)	32(62.7%)		
Lower	24(23.5%)	13(25.5%)	11(21.6%)		

PSM, propensity score matching; nICT, neoadjuvant immunochemotherapy; nCRT, neoadjuvant chemoradiotherapy; SMD, standardized mean difference; BMI, body mass index; CCI, Charlson Comorbidity Index; FVC, forced vital capacity; FEV1, forced expiratory volume in 1 second; DLCO, diffusing capacity of the Lung for carbon monoxide; EF, ejection fraction; ASA, American Society of Anesthesiologists Physical Status Classification.

Table S2. Baseline characteristics of the patients after OW.

	Overall	nICT	nCRT	P value	SMD
Variables	(n=39.8)	(n=19.9)	(n=19.9)		
Age (years)	72.74±2.45	72.74±2.66	72.74±2.24	>0.999	<0.001
Male gender	33.7(84.6%)	16.8(84.6%)	16.8(84.6%)	>0.999	<0.001
BMI (kg/m^2)	23.06±2.66	23.06±2.60	23.06±2.74	>0.999	<0.001
Smoking history	17.3(43.4%)	8.6(43.4%)	8.6(43.4%)	>0.999	<0.001
Drinking history	16.1(40.4%)	8.0(40.4%)	8.0(40.4%)	>0.999	<0.001
Family history of esophageal cancer	4.8(12.1%)	2.4(12.1%)	2.4(12.1%)	>0.999	<0.001
CCI score	6.00(6.00-7.00)	6.00(6.00-7.00)	6.00(6.00-7.00)	0.909	<0.001
Neutrophil($\times 10^9$/L)	2.95(2.49-3.83)	3.02(2.52-4.02)	2.92(2.40-3.79)	0.481	<0.001
Lymphocyte($\times 10^9$/L)	1.06(0.79,1.25)	1.10(0.79,1.25)	0.99(0.79,1.25)	0.669	<0.001
Platelet($\times 10^9$/L)	164.56(131.00-186.91)	168.17(131.00-194.78)	156.42(130.39-179.34)	0.379	<0.001
Hemoglobin(g/L)	117.57±16.06	117.57±15.56	117.57±16.68	>0.999	<0.001
Albumin(g/L)	37.39±3.78	37.39±4.04	37.39±3.53	>0.999	<0.001
FVC(L)	2.82±0.59	2.82±0.64	2.82±0.54	>0.999	<0.001
FVC(% pred)	89.70(81.82-104.40)	89.70(78.45-105.64)	89.57(82.06-101.09)	0.919	<0.001
FEV1(L)	2.21±0.45	2.21±0.50	2.21±0.39	>0.999	<0.001
FEV1(% pred)	93.36(84.00-103.46)	94.20(81.36-101.91)	92.80(85.75-103.53)	0.999	<0.001
FEV1/FVC(% pred)	79.18(73.06-84.60)	78.99(75.58-83.95)	80.87(71.95-84.68)	0.890	<0.001
DLCO(L)	6.11±1.49	6.11±1.47	6.11±1.54	>0.999	<0.001
DLCO(% pred)	83.90(74.12-94.70)	83.90(75.53-91.64)	79.28(70.61-94.70)	0.776	<0.001
EF(%)	67.72(65.00-70.00)	67.37(65.00-70.00)	67.68(65.00-70.00)	0.960	<0.001
ASA	2.12±0.34	2.12±0.36	2.12±0.33	>0.999	<0.001
Clinical T stage				>0.999	<0.001
2	0.6(1.5%)	0.3(1.6%)	0.3(1.3%)		
3	31.8(80.0%)	15.8(79.6%)	16.0(80.3%)		
4	7.4(18.6%)	3.7(18.8%)	3.7(18.4%)		
Clinical N stage				>0.999	<0.001
0	0.2(0.4%)	0.2(0.8%)	0.0(0.0%)		

1	16.4(41.2%)	8.1(40.8%)	8.3(41.6%)		
2	20.3(50.9%)	10.0(50.4%)	10.2(51.4%)		
3	3.0(7.5%)	1.6(7.9%)	1.4(7.0%)		
Clinical TNM stage				>0.999	<0.001
Ⅱ	0.5(1.2%)	0.5(2.5%)	0.0(0.0%)		
Ⅲ	30.6(76.9%)	14.8(74.4%)	15.8(79.3%)		
Ⅳ	8.7(21.9%)	4.6(23.1%)	4.1(20.7%)		
Tumor location				0.947	<0.001
Upper	6.4(16.2%)	3.4(17.0%)	3.0(15.3%)		
Middle	23.4(58.7%)	11.3(56.9%)	12.0(60.4%)		
Lower	10.0(25.2%)	5.2(26.1%)	4.8(24.3%)		

OW, 1. forced expiratory volume in 1 second; DLCO, diffusing capacity of the Lung for carbon monoxide; EF, ejection fraction; ASA, American Society of Anesthesiologists Physical Status Classification.

Table S3. Baseline characteristics of the patients after IPTW.

	Overall	nICT	nCRT	P value	SMD
Variables	(n=248.4)	(n=114.9)	n=(133.5)		
Age (years)	72.85±2.34	72.87±2.49	72.83±2.23	0.934	0.018
Male gender	214.1(86.2%)	96.9(84.3%)	117.2(87.8%)	0.578	0.035
BMI (kg/m²)	23.21±2.86	22.98±2.54	23.40±3.11	0.468	-0.143
Smoking history	107.7(43.4%)	43.9(38.2%)	63.8(47.8%)	0.388	0.096
Drinking history	99.6(40.1%)	44.1(38.4%)	55.6(41.6%)	0.767	0.033
Family history of esophageal cancer	28.0(11.3%)	10.9(9.5%)	17.1(12.8%)	0.587	0.033
CCI score	6.00(6.00-7.00)	6.00(6.00-7.00)	6.00(6.00-7.00)	0.920	-0.067
Neutrophil(×10⁹/L)	3.02(2.50-3.92)	3.03(2.56-4.17)	2.92(2.38-3.78)	0.179	0.116
Lymphocyte(×10⁹/L)	1.02(0.78-1.25)	1.08(0.81-1.26)	0.95(0.71-1.24)	0.368	0.122
Platelet(×10⁹/L)	165.29(131.00-194.19)	179.01(131.41-197.61)	156.78(129.33-178.84)	0.117	0.139
Hemoglobin(g/L)	119.23±16.34	118.55±16.19	119.82±16.58	0.765	-0.088
Albumin(g/L)	37.30±3.82	37.15±4.21	37.44±3.47	0.759	-0.079
FVC(L)	2.82±0.59	2.83±0.63	2.81±0.55	0.905	0.025
FVC(% pred)	88.93(81.31-103.70)	89.40(79.82-106.40)	88.53(81.84-100.32)	0.735	0.115
FEV1(L)	2.20±0.45	2.22±0.50	2.18±0.40	0.706	0.070
FEV1(% pred)	92.80(83.34-103.43)	96.62(82.85-107.90)	88.70(83.06-99.10)	0.376	0.209
FEV1/FVC(% pred)	79.18(72.96-84.32)	79.04(75.64-83.32)	79.21(71.71-84.61)	0.862	0.072
DLCO(L)	6.02±1.47	6.04±1.36	6.00±1.56	0.910	0.021
DLCO(% pred)	83.87(73.50-94.06)	83.90(75.90-91.20)	80.51(68.08-94.70)	0.687	0.039
EF(%)	68.00(65.00-71.00)	68.00(65.15-70.16)	68.00(65.00-71.00)	0.648	0.087
ASA	2.13±0.36	2.14±0.39	2.12±0.33	0.805	0.072
Clinical T stage				0.947	0.020
2	3.8(1.5%)	1.5(1.3%)	2.3(1.7%)		
3	200.7(80.8%)	92.9(80.9%)	107.8(80.8%)		
4	43.9(17.7%)	20.5(17.9%)	23.4(17.5%)		
Clinical N stage				0.664	-0.109
0	1.2(0.5%)	1.2(1.0%)	0.0(0.0%)		
1	95.8(38.6%)	47.2(41.1%)	48.6(36.4%)		
2	131.3(52.9%)	57.3(49.8%)	74.1(55.5%)		
3	20.0(8.0%)	9.2(8.0%)	10.7(8.0%)		
Clinical TNM stage				0.160	0.014
Ⅱ	2.7(1.1%)	2.7(2.3%)	0.0(0.0%)		
Ⅲ	192.6(77.5%)	85.9(74.7%)	106.7(80.0%)		
Ⅳ	53.1(21.4%)	26.4(23.0%)	26.7(20.0%)		
Tumor location				0.997	-0.011
Upper	35.2(14.2%)	16.6(14.5%)	18.6(13.9%)		

Middle	151.5(61.0%)	69.9(60.8%)	81.6(61.2%)
Lower	61.6(24.8%)	28.4(24.7%)	33.2(24.9%)

IPTW, inverse probability of treatment weighting; nICT, neoadjuvant immunochemotherapy; nCRT, neoadjuvant chemoradiotherapy; SMD, standardized mean difference; BMI, body mass index; CCI, Charlson Comorbidity Index; FVC, forced vital capacity; FEV1, forced expiratory volume in 1 second; DLCO, diffusing capacity of the Lung for carbon monoxide; EF, ejection fraction; ASA, American Society of Anesthesiologists Physical Status Classification. .

Table S4. The logisitc regression analysis of postopertive complications after matching/weighting.

		After PSM				After OW				After IPTW			
Complications	Treatment	Crude OR(95% CI)	P value	Adjusted OR(95%CI)	P value	Crude OR(95% CI)	P value	Adjusted OR(95%CI)	P value	Crude OR(95% CI)	P value	Adjusted OR(95%CI)	P value
All complications													
	nCRT	Reference				Reference				Reference			
	nICT	0.44(0.19-1.03)	0.059	0.37(0.12-1.13)	0.081	0.44(0.21-0.92)	**0.029**	0.32(0.12-0.82)	**0.017**	0.43(0.19-0.99)	**0.048**	0.34(0.12-0.94)	**0.038**
Major all complications													
	nCRT	Reference				Reference				Reference			
	nICT	0.47(0.18-1.19)	0.111	0.45(0.14-1.42)	0.173	0.61(0.28-1.36)	0.230	0.49(0.20-1.20)	0.119	0.58(0.24-1.40)	0.229	0.51(0.18-1.43)	0.201
Pulmonary complications													
	nCRT	Reference				Reference				Reference			
	nICT	0.32(0.12-0.85)	**0.023**	0.17(0.03-0.82)	**0.027**	0.31(0.13-0.78)	**0.013**	0.26(0.07-0.88)	**0.031**	0.34(0.12-0.97)	**0.044**	0.30(0.11-0.79)	**0.015**
Major pulmonary complications													
	nCRT	Reference				Reference				Reference			
	nICT	0.32(0.10-0.97)	**0.044**	0.26(0.07-1.07)	0.062	0.39(0.13-1.13)	0.082	0.34(0.10-1.19)	0.091	0.42(0.13-1.39)	0.156	0.24(0.04-1.54)	0.134
Anastomotic complications													
	nCRT	Reference				Reference				Reference			
	nICT	0.39(0.10-1.61)	0.195	0.19(0.03-1.48)	0.113	0.74(0.24-2.29)	0.606	0.44(0.13-1.55)	0.203	0.67(0.23-1.99)	0.476	0.51(0.15-1.73)	0.280
Major anastomotic complications													
	nCRT	Reference				Reference				Reference			
	nICT	0.47(0.11-1.99)	0.304	0.34(0.04-2.99)	0.333	0.9(0.30-2.72)	0.849	0.70(0.19-2.53)	0.585	0.77(0.26-2.27)	0.641	0.68(0.21-2.15)	0.509
Other complications													
	nCRT	Reference				Reference				Reference			
	nICT	2.66(0.49-14.41)	0.256	6.06(0.23-16.39)	0.281	3.94(0.80-19.36)	0.091	4.70(0.36-31.92)	0.239	3.22(0.78-13.32)	0.106	3.36(0.79-14.37)	0.102
Major other complications											.		
	nCRT	Reference				Reference				Reference			
	nICT	1.53(0.24-9.57)	0.649	3.63(0.43-30.98)	0.238	3.22(0.62-16.62)	0.163	2.86(0.53-15.47)	0.222	2.37(0.53-10.58)	0.259	2.59(0.51-13.25)	0.252
Pneumonia													

	nCRT	Reference				Reference				Reference			
	nICT	0.22(0.04-1.09)	0.064	0.23(0.03-1.91)	0.174	0.18(0.04-0.89)	**0.035**	0.17(0.01-2.33)	0.183	0.15(0.03-0.66)	**0.012**	0.15(0.02-1.47)	0.103
Anastomotic leakage													
	nCRT	Reference				Reference				Reference			
	nICT	0.38(0.07-2.03)	0.256	0.48(0.01-17.94)	0.691	0.61(0.17-2.27)	0.463	0.37(0.11-1.29)	0.119	0.53(0.15-1.90)	0.330	0.43(0.12-1.49)	0.181

nICT, neoadjuvant immunochemotherapy; nCRT, neoadjuvant chemoradiotherapy; PSM, propensity score matching; OW, overlap weighting; IPTW, inverse probability of treatment weighting.
